# A systematic review of qualitative studies exploring how parents affected by intimate partner violence and abuse and their children experience child welfare, health and criminal justice responses

**DOI:** 10.1186/s12889-026-26838-y

**Published:** 2026-03-07

**Authors:** William McGovern, Deborah Smart, Hayley Alderson, Carrie Barron, Victoria Cooling, Hilda Frost, Eileen Kaner, Simon Hackett, Ruth McGovern

**Affiliations:** 1 School of Communities and Education: Faculty of Health and Wellbeing, Northumbria University, Coach Lane Campus, Benton, City of Newcastle, Tyne and Wear NE7 7QA UK; 2 Population Health Sciences Institute, Newcastle University, Baddiley-Clark Building, City of Newcastle, Tyne and Wear UK; 3 Office for Health Improvement and Disparities, Northumbria Healthcare NHS Foundation Trust, Goldcrest Way, Newburn Riverside, City of Newcastle, Tyne and Wear UK

**Keywords:** Intimate partner violence, Parents, Systematic review, Qualitative studies

## Abstract

**Background:**

Parental Intimate Partner Violence and Abuse (IPVA) is a complex issue, which requires a sensitive response from a range of services. This review aimed to identify and synthesise qualitative research examining the perceptions and experiences of parents and children affected by IPVA and their interactions or engagement with various child welfare, health and legal systems and services.

**Methods:**

We conducted a systematic review of the international literature, searching 11 electronic databases from inception to November 2023 and supplemented this with a grey literature search. Studies were included if they provided qualitative accounts from adult and/or child victims/survivors of IPVA and/or adult perpetrators reporting on experiences of child welfare, health, and/or criminal justice intervention.

**Results:**

A thematic synthesis of 39 individual studies (38 papers and 1 book chapter) which include the perspectives of (n-825) mothers/adult females (n-107) children and (n-58) fathers was undertaken. Three overarching themes were identified: (1) the importance of supporting the family whilst safeguarding the child (2) systems failing of services to hold the perpetrator to account and (3) systems that retraumatize the Family.

**Conclusions:**

Services should provide a whole-family approach, which responds to the needs both the parent and child victim/survivor, and recognises the parental identity of the perpetrator. Interventions with adult victims/survivors should take a strengths-based approach, whilst holding the perpetrator to account. Particular care is needed when families are involved in family court to avoid re-traumatisation.

**Supplementary Information:**

The online version contains supplementary material available at 10.1186/s12889-026-26838-y.

## Background

Parental intimate partner violence and abuse (IPVA) is a substantial public health challenge [118] and child protection concern [48, 52]. Women who experience IPVA are at increased risk of injury [119] and death [1], and are more likely to develop a range of mental health problems, experience suicidal ideation, and attempt suicide [86]. In addition to the significant harms of IPVA to the adult victim, there is well-established evidence documenting the harmful affect upon children throughout their life course, following exposure to parental IPVA [75]. Pre-natal exposure increases the risk of miscarriage [53], low birth weight of the infant [53] and neonatal death [3]. Post-birth, parental IPVA has also been found to have a substantial impact children [56] resulting in the Domestic Abuse Act in the UK recognising children as victims regardless of whether they are present during incidents of abuse. The harms resulting from IPVA on children will be dependent on a range of risk and protective factors which include developmental stage, gender, maternal mental health and quality of parenting [37]. However, these children are more likely to suffer ill-health and not access appropriate care for their health needs [8], have lower educational performance [21], resulting in poor life chances [8] and often experience mental health problems in childhood [2, 86], including internalising and externalising problems, and experience childhood trauma symptoms [33, 41], which often persisting into adulthood [101, 102]. Further, they are more likely to themselves be an adult victim or perpetrator of IPVA [78, 113]. Parental IPVA has been found to negatively affect the structures and functions of the family and the relationship between the non-abusing parent and the child [10].

Addressing IPVA is an international priority and across different nations several intervention approaches have been designed, implemented, and evaluated. IPVA interventions are complex interventions. They may be primary, secondary or tertiary interventions which are designed to prevent IPVA from occurring, to respond to the immediate effects and to protect or mitigate its long-term impacts (Home Office, 2023). There have been multiple systematic reviews which have examined the effectiveness of a range interventions including those that aim to prevent IPVA on a primary [77, 115], secondary [60, 77] and tertiary level [60, 77], increase detection [24, 34, 82] and promote recovery [11]. Reviews have examined interventions delivered within a range of settings including schools and colleges [77, 115], health [34, 109], child welfare [57, 83, 84] and criminal justice [61, 91, 116] and studies conducted in low and middle income countries [60]. Further reviews of tertiary interventions have examined effectiveness at addressing the mental health impact upon the adult victims/survivors [55, 83, 84, 97, 103] and child victims/survivors [49, 98, 112]. Whilst these reviews provide important insights into intervention effectiveness, they are typically individualistically focused and fail to consider the complex systems relating to the family and the child welfare, health, and criminal justice services, in which help-seeking and intervention occur. Reviews also fail to fully recognise or consider concerns with intersectionality, first termed as intersectional feminism [25] and the ways in which how different groups of women are affected by concerns with IPVA and the ways in which identity is linked to further forms on inequality and discrimination.

Parents and children who experience IPVA have a range of complex and sometimes competing problems. They often require interventions from multiple services and are required to navigate different types of services and systems, including child welfare, health, criminal justice and family courts. The potential for these services to cause harm to the mother and the child and their wellbeing has previously been documented [104]. In addition Mothers often also face “no win situations” when accessing services to support themselves and their children. If mothers stay in a relationship they are accused of “failing to protect” and if they leave or seek support they face increased risk of stalking, harassment or abuse (Saunder and Oglesby, 2016). In addition, the exposure of children to IPVA may result in safeguarding procedures being implemented, including statutory responsibilities to protect or remove children from situations of significant harm resulting in tensions between safeguarding the child and supporting the adult victim [46]. Interactions with practitioners who directly provide or signpost to interventions can be experienced as threatening by parents, resulting in their resistance to engage with available care [38]. The way in which parents and children affected by parental IPVA experience child welfare, health and criminal justice services is likely to impact upon their engagement with these services and their effectiveness at reducing IPVA and its impact. There has been a number of studies which have sought to understand how parents and/or children who are victims/survivors of IPVA experience services which intervene with families, and a smaller number examining the service usage experience of parents who perpetrate IPVA. Typically, these studies have focussed on the experiences of mothers and children as they engage with individual types of services or reported on traditional settings like refuges, counselling, child welfare and criminal justice. However, to date, no review has engaged with the breadth of this literature, across the highly differentiated and wider services and settings that support individuals and families.

### Study aim and objectives

Our review systematically searched and reviewed the international literature to examine how parents and children affected by IPVA experience child welfare, health, and criminal justice responses. In doing so, we sought to produce a family-focused account and answer the following questions:

- What are the perspectives and experiences of parents and children who are victims/survivors of IPVA of child welfare, health, and criminal justice responses?
- What are the perspectives of parents who perpetrate IPVA of child welfare, health, and criminal justice responses?
- How do the experiences of multi-agencies differ depending upon agency type?

## Methods

This review is reported according to the guidelines of the “Preferred Reporting Items for Systematic Reviews and Meta-analysis” (PRISMA) statement [87] and the review protocol is registered with PROSPERO (CRD42020213980). We searched the international literature from inception to November 2023 using electronic databases Medline (OVID), PsycINFO (OVID), CINAHL (EBSCO), SCOPUS, Applied Social Science Index and Abstract (ProQuest), International Bibliography of Social Science (ProQuest), ProQuest Criminal Justice (ProQuest), ProQuest Social Science Journals (ProQuest), ProQuest Sociology (ProQuest), Social Service Abstracts (ProQuest), Sociological Abstracts (ProQuest). No date or language restrictions were applied. A search strategy using mesh terms, thesaurus headings, Boolean and proximity operators was adapted for each database and implemented. The full search strategy is available within the supplementary file.

We followed guidance on reducing the risk of publication bias in qualitative systematic reviews [90]. This included searching for conference abstracts to identify studies which may not have been published and supplementing this by searching websites of relevant organisation for grey literature. Organisational websites included Victim Support, Survivors Trust, National Institute for Health and Care Excellence (NICE), National Society for the Prevention of Cruelty to Children (NSPCC).

### Review inclusion criteria

All titles and abstracts were screened for inclusion by a minimum of two reviewers (WM, RM, DS, HA, SH, HF, CB), using pre-specified inclusion and exclusion criteria, retrieving full papers for all potentially eligible studies and evaluating in full text. Discrepancies at each stage were resolved by discussion or by consulting a third reviewer if consensus could not be reached. Studies using qualitative methods reporting primarily narrative data were included if they met the following criteria:

- the sample consisted of parents (mothers and/or fathers including step and foster parents) or their children (aged 0–18 years). The review also included pregnant women and their partners.
- provided accounts from adult and/or child victims/survivors of IPVA and/or adult perpetrators reporting on experiences of being supported or managed by child welfare, health, and criminal justice services. This includes statutory and non-statutory interventions, services and care provided in support of the parent (victim/survivor and/or perpetrator) or the children, to safeguard or to prosecute criminal behaviour.

### Quality appraisal

The quality of the included qualitative studies were assessed using the Critical Appraisal Skills Programme (CASP) tool, as recommended by Cochrane Guidance [80]. The CASP checklist is the most widely used appraisal tool for qualitative studies [63] and presents 10 questions about study design, findings and transferability of results and there was a two-stage process which guided the quality assessment process [15]. All papers were reviewed and assessed against the CASP checklist for quality, whilst being reviewed and assessed for relevance [28]. The studies were categorised according to quality and relevance [76] either as:

(A) a key paper that was most relevant and conceptually rich, with no or few quality issues
(B) a secondary paper, that was relevant but with limited themes and data, and/or some quality issues
(C) satisfactory, that was less relevant to the review and/or the CASP appraisal highlighted major limitations related to the quality of reporting

In accordance with guidance, quality assessment outcomes were not considered a reason to exclude studies. Rather, the quality appraisal results were used to inform the synthesis and instruct our thematic analysis, [63].

### Data analysis and synthesis

Data were extracted by two reviewers using a bespoke data extraction template, which included: study type, sample and participant characteristics, analytical approach and findings relevant to the review questions. The data synthesis in this review was informed by [110] three stage thematic method [110]. Data analysis was an iterative process. Firstly, we read all key texts (CASP A) and coded these line by line. Within the second stage of analysis, we generated descriptive codes from the coded key texts. and applied these to data extracted from our secondary papers (CASP B). Where it was deemed appropriate and meaningful in relation to our research questions, we created new codes based on secondary papers, which were subsequently applied to key papers. The remaining lower quality papers (CASP C) were used for confirmatory coding only. Within the final stage, broad and high-level themes were extracted which related to the study aims and objectives and “framed” into Microsoft word documents in relation to the reported “positive and negative” experiences of respondents in studies [96]. These broad themes were re-read and from them, we developed a more detailed set of descriptive themes that were categorised, collated, and recorded on Microsoft word documents. High-level themes were then formally presented, discussed and refined until a fully drafted set of analytical themes were generated [110]. Agreement was reached in each stage of thematic analysis by discussion within the review team. Table 2 details the descriptive and analytical themes and the papers contributing evidence.

## Results

**Fig. 1:**
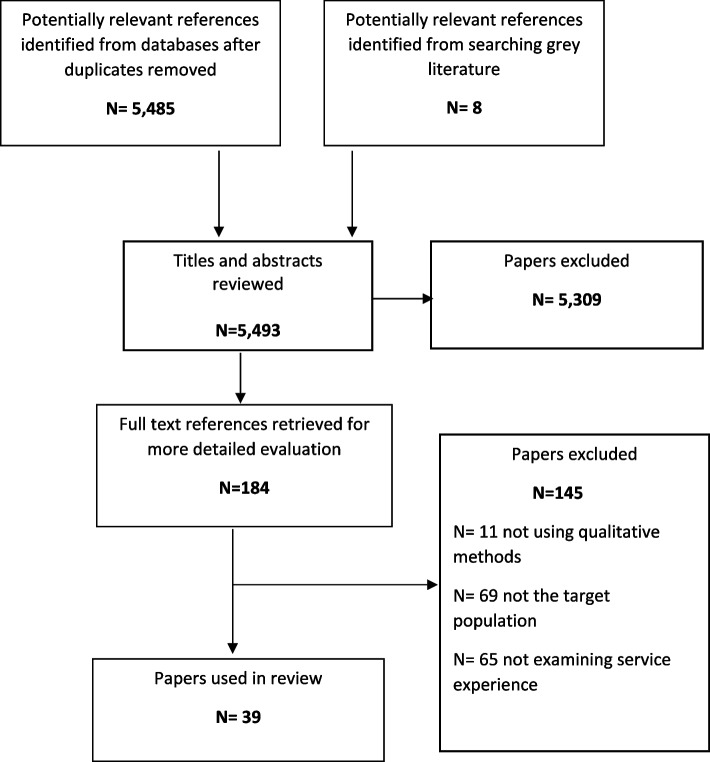
Flow of studies

Our search identified 5,490 potentially relevant references. Of those, following title and abstract screening, 184 full papers were retrieved. Thirty-nine papers each reporting unique studies met our inclusion criteria and were included in our review (Fig. 1).

### Description of studies

The 39 studies had a total sample of 990 participants, the majority of which were adult female victims/survivors (*n* = 825). There were also 107 child victims/survivors and 58 perpetrators. All but one of the participants who were perpetrators of IPVA were adult male. Of the 39 studies, 15 examined child welfare services including family courts, 6 examined multi-agency responses, 3 healthcare (2 GP and 1 Antenatal) 5 housing/refuge/emergency shelters/transitional housing, 8 structured psychosocial interventions and 2 criminal justice services. The greatest proportion of studies were conducted in the US (16; 41%), a further 9 in the UK, 6 in Australia, 4 Sweden, 2 Canada, 1 Nepal and 1 Bhutan. Most studies reported recent research; 30 (77%) were conducted within the last 10 years and 16 (41%) in last 5 years (Table 1).

**Table 1:** Extended description of studies

Study Details	Setting	Research Focus	Sample and Analysis	CASP	Research Themes Located in Literature
Anitha, (2010) [4] UK	Adult Social Services	How Policy and Practice affects mothers in Adult Social Care	30 refugee Mothers Semi Structured Interviews and thematic analysis	B	1
Antle, (2010) [5] USA	Social Welfare	Effects of mandatory reporting of DV on Victims and children	24 Mothers Structured telephone Interviews	B	1
Baccus, (2003) UK	Health Maternity Care Services	Help seeking behaviours of pregnant women in health care	16 Mothers, Age 18–36, Semi Structured interviews and content analysis	A	1
Berger, (2008) [12] USA	Adult Social Care	The effects of Law Guardians on mothers’ wellbeing	10 Mothers Unstructured Interviews and thematic analysis	B	1 and 2
Bostock, (2009) [13] UK	Adult Social Care	Social processes and women’s experiences of IPVA services	12 Mothers, Age 21–56 Unstructured Interviews and grounded theory	A	2
Bowstead, (2019) [14] UK	Adult Social Care	Refuge services as places of safety and recovery	20 Mothers, Age 18–56 and 8 Practitioners interviews and group work	C	1
Choden, (2021) [18]	Social Care Therapeutic	Barriers mother face accessing services	15 Mothers, Age 25–40, semi structured interviews and Thematic Analysis	A	2
Clark, (2018) [20] USA	Transitional Housing (TH)	Examination of experiences and needs in relation to TH	30 Mothers, Age 22–58 semi structured interviews Thematic Analysis	B	1
Clough, (2014) [22] USA	Transitional Housing	Explored abused mothers’ experiences of accommodation	11 Mothers, Age Av: 32 years old and semi structured interviews, thematic analysis	A	2 and 3
Devoe, (2003) [27] USA	Multiple Services	Barriers to service delivery for Battered Mothers and Children	43 Mothers, Age 20–30’s, Interviews and Focus Groups and thematic analysis	B	1 and 2
Douglas, (2018) [30] AUS	Criminal Justice	Effects of legal engagement on Mothers Health and WB	65 Mothers, Age 23–68 semi structured interviews and thematic analysis	A	3
Earner (2009) [31] USA	Social Welfare	Mothers experiences of accessing public child welfare	19 Mothers, Age 25–66 interviews and focus groups and unspecified analysis	B	1 and 2
Fisher (2019) [35] USA	Sheltered Accommodation	Why Mothers disengaged with services	34 Mothers, Semi Structured interviews and grounded theory	B	1 and 2
Fleury-Steiner, (2011) [36] USA	Shelters, Advocacy Programmes Counselling and CJS	Impact of Mothers engagement with Judicial System and Child Protection Services	19 Mothers, Age 21–45 interviews with open and closed questions and unspecified analysis	A	2 and 3
Grillo, (2019) [40] USA	Health Care	Perceptions of trauma informed intervention for IPV	25 Mothers, Age 29–70 focus group and person-centred outcomes analysed	B	1
Gutozski, (2020) USA	Family Court	Mothers perceptions of seeking child custody via family court	19 Mothers, Age 34-67surveys and interviews and naturalistic thematic analysis	A	2 and 3
Herman (2019) [45] USA	Therapeutic Intervention	Experience and needs of Y/P in relation to IPVA	28 young people, 22 mothers, 6 fathers, Age 16–22 focus groups and Thematic Analysis	B	1 and 2
Hughes, (2016) [51] CANADA	Housing	Mothers involvement in Child Welfare Services	32, Mothers, unstructured interviews and narrative analysis	A	1 and 2
Hughes, (2012) [50] CANADA	Family Court	Mothers involved in Child protection and Family Law	35, Mothers interviewed, semi structured interviews and thematic analysis	A	2 and 3
Kamal, (2016) [54] SWI	Social Welfare	Experiences of Attachment based intervention for Parents	16 mothers, Av Age 38 and 10 fathers, Av Age 44. Focus groups and Thematic Analysis	C	1 and 2
Khaw, (2021) [58] USA	CJS and Family Court	Perceptions and experiences of custody determination	24 Mothers, Age 23–48 secondary analysis using Thematic Grounded Theory	A	2 and 3
Kiamanesh, (2019) [59] NORWAY	Housing	Mothers accessing a range of IPV services unspecified	18 Mothers Age 24–53 semi structured interviews informed by IPA	B	2
McGee, (2000) [67, 68] UK	CJS, Housing and Adult Social Care	Mothers and Childrens experiences of IPVA support	54 Children and 47 Mothers, semi structured interviews, analysis unspecified	C	1 and 2
McManus, (2013) [72] UK	Therapeutic	Impact of the program ability to achieve positive outcomes	11 Children Age 7–11 and 15 Mothers Age 27–41 semi structured interviews	B	1
Meyer, (2010) [73] AUS	CJS	Factors that influenced help seeking behaviours in Mothers	29 Participants, Mothers, age 21–62 in-depth interviews with thematic analysis	B	2 and 3
Molina, (2017) [74] USA	Adolescents Theraputic	Experience of mutual aid processes	8 male and 8 female, Age 18–19 adolescents, grounded theory, focus groups and analysis	B	1
Narula, (2012) [79] USA	Health	Perceptions and experiences of accessing GP for IPV and care	10 Mothers, Age 40–73 Semi structured interviews and thematic analysis	A	1
Onsjo, (2023) [85] SWE	Children’s Mental Health	Helpful/hindering aspects of multiple services	13 Female/4Male, Age12-25 Semi Structured interviews with IPA Thematic Analysis	A	1
Pearson, (2022) [88] UK	CJS and Therapeutic	Experiences of treatment and perceptions benefits/outcomes	13 Males/1 Female, Av Age 33.1. semi structured interviews Thematic Analysis	A	1 and 2
Pernebo, (2016) [89] SWE	Therapeutic Group Program	Experiences of psyo-educational group treatment for IPV	9 Children, 5 girls, 4 boys, Age 4–6 years, IPA informed interviews and analysis	A	1
Rathus, (2019) [92] AUS	Familycourt	Experiences of mothers during report writing process	10 Mothers, Age 28–46, Interviews and thematic analysis	A	2 and 3
Renner, (2022) [93] USA	Adult Social Care	Exploring mothers experiences and psycho-ed outcomes	15 Mothers, Age 27–48, survey and semi structured interviews, thematic analysis	A	2
Robbins, (2018) [99] UK	Adult Social Care	Trust in relation to Social Work and Child Protection	26 Mothers, focus group and thematic analysis	C	1, 2 and 3
Roberts, (2015) [100] AUST	CJS and Family Court	Psychological impact of Mothers engagement with Family Court	15 Mothers, Age 25–56, Face to face and telephone interviews, narrative analysis	B	3
Rishal, (2016) [95] NEPAL	Adult Social Care	Perceptions and experienced of ante natal care	12 Mothers, aged 22–45 in depth interviews and thematic analysis	A	1 and 2
Smith, (2019) [105] AUST	Adult Social Welfare	Experience of consequence and accountability for families and formal interventions	20 Fathers, 15 women and 7 practitioners, interviews and thematic analysis	B	1
Stephens, (2022) [106, 107] USA	Therapeutic	Relationship with children during intervention	14 Fathers, Age 18 +, focus groups and interviews, grounded and thematic analysis	A	1 and 2
Stewart, (2023) [108] UK	Childrens Services	Experience and Impact of involvement with SWCP	19 Mothers, Age 18–47 semi structured interviews and thematic analysis	B	1 and 2
Wendt, (2013) [114] AUST	Refuge and Transitional Housing (TH) Support	Experiences and outcomes for mothers and children in supported and TH	13 mothers, Age 18 + semi structured interview and thematic analysis	A	1

### Themes

We identified three overarching analytical themes. These are Theme ne: The importance of supporting the family whilst safeguarding the child; Theme Two Systems failing to hold the perpetrator to account and Theme Three: Systems: re-traumatising the family. The evidence informing each theme is discussed narratively and an overview of each study’s contribution to the descriptive and analytical themes are included in Table 2. In the findings which are presented below we provide direct quotations from studies in the review to illustrate the data and lived experience of those family members accessing IPVA support services.

**Table 2:** Themes and codes; identification and significance within studies

Themes and Codes	Location of Codes and Saturation within Review Literature/Studies
Theme One:	The importance of supporting family whilst safeguarding children
Providing emotional and practical support and information	[5, 14, 20, 22, 35, 40, 45, 51, 72]
Interpersonal practitioner skills	(Baccus, Meze et al, 2003), [31], (Monila and Chapple, 2017), [20, 40, 45, 67, 68, 88, 95, 99]
The importance of recognizing and responding to different family members needs	(Monila and Chapple, 2017) [26, 36, 40, 50, 67, 68, 85, 88, 89, 105, 114]
The importance of recognizing and responding to different family members status and roles	(Erikson, 2009) (Nurala, Agarwal et al, 2012) [4, 12, 45, 54, 72, 88, 105–107]
Theme Two:	Systems failing to Hold Perpetrator to Account
Questioning Mothers Character, Abilities and Choices	[12, 13, 18, 27, 45, 73, 92, 95]
Directly Blaming Mothers	[18, 43, 50, 58, 73, 99, 100]
Not involving Family members in key decision making	[18, 22, 51, 59, 72, 93]
Displacing focus or responsibility onto individual family members	(Earner, 2019), [35, 36, 59, 92, 95]
Theme Three	Systems that Retraumatise the Family
Sharing Spaces and contact with ex-partners	[30, 43, 50, 73, 92]
Being coerced and failing to protect family members in legal contexts	[22, 30, 36, 43, 58, 73, 106, 107]
Failing to support mothers’ recovery and keep them and children safe	[43, 58, 99, 100]

## Results

### Theme one: the importance of supporting the family whilst safeguarding the child

Within the included studies, practices were identified that could be built upon to potentially improve the perceptions and experiences of families as they engage with IPVA services and systems. Those services with systems which listened to, considered, recognised and responded to the concerns and support needs of the family unit (and its individual members) were experienced positively by parents and children affected by IPVA. Typically, the services within these studies utilised a family focused approach [72] or provided specialist services in response to the needs of mothers [14, 40, 67, 68, 114] fathers [88] and the child [67, 68, 74, 89]. Mothers, like Kate in the study below, reported that when they were effectively supported by services, they felt enabled to focus their attention on supporting their children’s needs:

“it took a big load off me…..and they are like “that’s ok, we will do what we can to help you. All you got to do is be a mother to your children and concentrate on them” That took a lot of load off” (Kate; cited in, [114]:521).

Within the studies, mothers reported benefitting from the provision of practical advice and guidance in response to their experiences of IPVA [5, 20, 35]. This included onward signposting into employment, educational, transitionary housing, and community services [114]. Mothers described feeling empowered when presented with choices, and information about available services, and being able to make informed decisions about and act on their own needs without support from professionals [14, 40]:

“before I came here, I did not know where to go or who to get in contact with and stuff like that and since I’ve been here I’ve been able to use a lot of services because of the networks they have” (Essie, cited in, [114]: 522).

The positive attributes of individual professionals such as being sensitive, genuine and caring [40, 74, 114] and non-judgemental [20] were highlighted as necessary in helping mothers [99] and fathers [88] to engage and then stay engaged in different services [67, 68].

Studies reported that services often overlooked the parenting responsibilities of fathers who had perpetrated violence and instead intervened with the abusive behaviour within the context of the intimate relationship. This resulted in their identity and role as a father being dissembled and replaced with that of perpetrator and abuser [106, 107]. Overall, studies suggested a motivational benefit in acknowledging the perpetrator’s role as a father when providing IPVA interventions [45, 54, 105]. When intervened with within a family context, father’s self-reported an increased ability to communicate with their (ex)partner and children, refrain from using controlling behaviours, maintain a healthy relationship with their (ex)partner and improve their parenting practices [54]. Studies also reported that activities and conversations designed to enable fathers to reflect upon their behaviour as a paternal risk factor in both mandated and non-mandated perpetrator programmes provided fathers with the opportunity to acknowledge the potentially negative characteristics they possess and to reframe their understanding of their use of IPVA [88, 106, 107].

Studies which examined adolescent children’s experiences of individual counselling [85] and mutual aid support groups (Molina 2017) as co-survivors following exposure to parental IPVA reported that being recognised as a victim/survivor of IPVA was seen as an overwhelmingly positive experience for children. These studies also highlighted that a proportion of children continue to be exposed to IPVA and may experience ongoing and enhanced feelings of insecurity whilst accessing support [85]. Despite this, children were found to benefit from an opportunity away from home where they could talk and be listened to, wherein they were allowed to work at their own pace with professionals [67, 68, 85]. This was found to lead to children experiencing improvements in social and emotional functioning [74].

“She [social worker] wouldn’t always talk about it straight away, like I would talk about everything, she would talk about how things were going and ask me what was happening. And like after a while, it built my trust up sort of thing, so when things did come up I found that I could talk to her about them more easily” (anonymous female in [67, 68]:, pg 84).

Conversely, child welfare services were often perceived to be focused upon only safeguarding the child. Mothers [59, 92, 95] and fathers [105–107] were often critical of child welfare services for failing to provide family support. Studies reported that mothers experienced professionals in in these settings to be disinterested in their IPVA related needs and that professionals were only concerned with fulfilling their duties to safeguard the child [12] and completing assessments [50, 95]. This resulted in missed opportunities to engage mothers in a meaningful dialogue about their experiences to understand the nature of their IPVA and respond to their needs as victims/survivors [67, 68]. A small number of studies examined the experiences of vulnerable populations of mothers including young mothers [45] and mothers who had been in care themselves as children [36, 51]. These mothers were reported to find it particularly difficult to have their voices heard, and their concerns responded to by child welfare services. Mothers attending health care settings for treatment for IPVA related concerns and injuries also reported negative experiences resulting from the ways in which clinicians would typically focus upon treating the presenting physical condition (such as pain, injury, ailments) and psychological symptom (depression, anxiety, sleeplessness) without recognising or enquiring about the cause [9, 95]. In these types of settings when mothers-initiated conversations about IPVA with health care practitioners, they reported that the practitioner often lacked empathy, did not offer support in response to their needs [95] and typically responded to them with rigid adherence to child safeguarding procedures wherein authorities were notified of the risk to the child [79].

The needs of children in services were often reduced to preventing further exposure to recurrence of IPVA incidents, with little recognition of the child’s needs or the longer-term impact of IPVA [4, 26, 67, 68]. At times, children were also perceived to be ‘too young’ to be impacted by their experiences and therefore received no support or were not afforded a voice when determining the safeguarding response [12, 32]. Similarly, four studies reported that fathers often felt their status as a father was ignored by children’s child welfare services, which they perceived resulted in them being excluded from important decisions about the child and their day-to-day lives [45, 54, 88, 105].

### Theme two: systems failing to hold the perpetrator to account

Many of the included studies reported on the harms that mothers perceived and experienced from the ways in which professionals blamed them for their own abuse, and rarely held their abuser to account [50, 100]. Rather, questions relating to the mother’s character [12, 27, 45, 73, 92, 95] and judgement about their lifestyle (e.g. using substances) and choices they made (e.g. delay in reporting IPVA or to remain in a relationship with the father) [13] were common. Further, mothers reported that they were often blamed for instigating [18], or accused of exaggerating, violence [58] and failing to protect their child from their abuser [43, 73, 99]. These blaming and often contradictory experiences lead to their suitability as mothers being questioned [12, 27, 73, 92], and in some instances, their child being removed from their care [51]. This overall “displaced focus” upon the mother’s perceived deficits and not upon the actions of the father, created fear within the mothers and a reluctance to engage with services [35, 36], [31].

“The [worker] didn’t really listen. They did not seem interested [in the domestic violence] they’d go back to whether or not I would support his relationship [with the daughter],why I had stopped contact. Probably of anything, that was the bulk of the discussion […] I stopped contact because he’d [father] had gone off [verbally abusive] at my friend and I and he was quite clearly out of it [high on drugs]. He [worker] was talking to me about not facilitating the relationship […] and that it was serious to withhold a child and it would damage Wendy’s relationship [with her father]. The [worker] did not see any reason why she could not have overnights with her father.” (Bronwyn: mother, in [92]:23).

Mothers within the studies described being required to engage with therapeutic parenting programmes as a result of being a victim/survivor of IPVA. A small number of mothers reported that they found benefit in learning about how IPVA may have impacted their children and the mother–child relationship [93] and that parenting programmes were at times a source of validation, resulting in the realisation they were already practicing as ‘good mothers’ [93]. However, the majority did not perceive parenting programmes to be relevant to their needs [22, 51] and were critical of the suggestion that they needed to attend parenting programmes designed to teach them parenting skills or how to better bond with their child [72].

“me and my son have a good bond anyway…..some parents may feel that, but me and [my son] don’t need to be playing daft games. (Mother, in McManus; pg 299).

The inappropriateness of the service responses was particularly highlighted in studies in which mothers were immigrants. One study identified that a lack of recognition in relation to their needs, experiences and situation resulted in them being offered or mandated to attend interventions that were both inappropriate and culturally insensitive [59]. Within studies examining the experiences of mothers from minoritized ethnic groups, parenting programmes were cited within studies as irrelevant and as an additional source of stress and harms because the course content lacked cultural relevance [18]. The mother in the example below highlighted that a parent course she was expected to attend failed to recognise her culture and values, leaving her feeling her parenting was criticised.

“The CPS (Crown Prosecution Service) sent me on a course. It was good, but most of the things discussed here I already knew. And we have a different parenting style and culture. We don’t interact with our children in the same way as in Norway. But that does not mean it is wrong […] So the course didn’t help much. It just stressed me” (Ghada, in, [59]; pg 304).

### Theme three: systems that re-traumatise the family

Studies reported that adult and child victims/survivors often found services to be retraumatising. In particular, family courts were reported to fail to take steps to protect the mother from the emotional trauma which occurred, as they were made to share physical spaces with the perpetrator of their abuse, such as court waiting rooms [30, 42]. Mothers also reported harms that resulted from the harsh and hostile questioning techniques that were used by court officials often causing emotional distress [12, 30, 43]. Studies reported that mothers and children often felt trapped and betrayed by services in family court settings as a result of being made to have contact with the child’s father within custody cases [43, 73, 100]. These studies found that mothers who had previously been required by children’s services to end all interactions with the child’s father for their children’s welfare and protection, were now required to face their abuser in family court. Mothers reported that they felt the “family court system was unfair” and described how they were “coached” into downplaying the extent of violence by their legal representatives to avoid being accused of “lying” or “coercing” their children into making false claims by the father’s representative [58]. Conversely, studies reported that fathers experienced both the criminal justice system and family courts to be a place where their guilt was assumed, and that they were often ostracized from their children. It was reported that fathers were more likely to accept a “plea bargain” [guilty] and agree to attend a “Batterers Intervention Programme” in lieu of a trial with the possibility of gaining a conviction and incarceration, despite frequent reluctance by fathers to personally accept wrong doing [106, 107].

“In exchange [for a plea (original emphasis)], they would just charge me with disorderly conduct. Which would not show up on my record […] And then when I went in the court and the judge asked me……….I just had to say yes to everything” (Focus group participant, [106, 107]).

Studies described mothers feeling vulnerable to further IPVA by court ordered child contact time which typically did not include safety planning for the mother and child. Mothers reported that unsupervised court-ordered contact time allowed abusive ex-partners the opportunity to continue to threaten, manipulate, intimidate, humiliate and degrade mothers [50, 92]. Mothers were often left to manage their own safety, the safety and support needs of their children and the interaction the father had with their children without any additional or ongoing support [99]. These types of experiences, and the further and ongoing exposure to trauma, affected the mother and child as individuals, and their ability to move on and recover from their experiences of IPVA [58]. Mothers and children often reported that they felt emotionally tired, let down having exited with custody and visitation arrangements that they felt left them open to further abuse and revictimized by family courts [58]. Moreover, women were left feeling revictimized by the experience [42].

“[I’m] being victimised by the very same system that is supposed to protect me” (Olivia, [42]:450).

## Discussion

Our systematic review is the first to synthesise parent and child experiences and perspectives of multi-sector responses to IPVA. It is clear from this review that child welfare, health and criminal justice services have the potential to strengthen the position of the family and individual family members but also to be a significant source of harm, particularly mothers and children as co-victims and co-survivors [56]. More specifically, this review has identified strengths within services and systems and that IPVA support that is family-focused is valued by adult and child victims/survivors and perpetrators alike. Such approaches were reported to provide validation for each family members’ experiences and role, as well as recognition of the impact of IPVA upon them and the family. However, our review found that this support was inconsistent and typically fragmented from wider provision. Parents and children in the studies included in our review typically described services which focus upon safeguarding the child, without necessarily supporting the family and attending to all of their needs. Within this was a tendency to focus upon perceived maternal deficits for failing to protect the child from the abuse of the father. This finding is consistent with previous research which has highlighted tensions between services which are typically either adult or child focused [46, 62, 66], and overlook the complex relationship between the safety of the mother and that of the child [49, 70, 117]. Additionally, the longer-term impact of IPVA upon the child was reported to be overlooked within services. The introduction of the Domestic Abuse Act (2021) in the UK marked the start of children being officially recognised as victims of domestic abuse. This is in response to significant evidence showing that child victims and survivors go on to experience physical [94] and mental health problems in adulthood [86, 101, 102] and are more likely themselves to be victim to, or perpetrate IPVA [113].

Very little is known about the actual needs, experiences and expectations of perpetrators as they engage with IPVA services, with most research focusing upon mothers [47]. However, findings from this review have reinforced the need for a ‘pivot to the perpetrator’ [64], wherein practice is reconfigured to acknowledge the impact of this paternal risk factor upon children [81] and hold fathers to account for their actions. Such an approach would mark a move away from the victim-blaming and ‘failure to protect’ discourse [7]. Mothers in particular should not be made to feel worse, inadequate as a parent or threatened by individual or organisational practices when having discussions and assessments made in relation to any types of safeguarding issue or concerns [64]. The importance of a strength-based perspective when working with mothers and children impacted by IPVA is highlighted [72] as necessary to facilitate social and emotional recovery [40]. Additionally, our review found evidence that fathers valued the opportunity to reflect upon and understand the implications of their abuse and how this interacts with their role as a father finding this could impact and motivate behaviour change.

Our review has highlighted the potential of services to re-traumatise adult and child victims/survivors of IPVA wherein victims/survivors were required to recall and relive traumatic experiences and/or have contact with the perpetrator of their abuse. These services were found to inhibit the ability of individuals and/or families to recover, and in some instances, revictimize adult and child survivors. It is clear that a trauma-informed approach is needed within all services working with adult and child victims/survivors of IPVA [16], wherein the impact of trauma is recognized and appropriately responded to through adherence to the six core principles: safety, trust, choice, collaboration, empowerment and cultural consideration [17]. Trauma-informed care has been found to significantly improve the psychological health of women who have experienced IPVA [19, 44] and provide a safer environment for victims/survivors and avoid system-orientated re-traumatization [6]. In all service settings this process and way of working calls for a combination of intuitive and skilful self-reflective individual practice from practitioners across the board. It also calls for increased organisational manoeuvrability in being able to centralise the management of safety concerns whilst holding neutrality and supporting the holistic needs and involvement of each and all family members [39]. This type of practice and manoeuvrability is required when supporting adult and child victims/survivors of IPVA and when fundamental decisions are made about the individual family members in relation to managing risk and safeguarding concerns alongside the welfare related needs of individual family members.

### Practice implications

This review has important implications for practice with parent and child victims/survivors and perpetrators of IPVA. Firstly, all services should take a whole family approach when working with families affected by IPVA wherein the recognise and respond to the needs of both the parent and child victim/survivor [11]. Such an approach is likely to require multi-agency wherein there is a co-ownership of a clear practice model [71] and cross-agency practitioner training [65]. Secondly, services should ‘pivot to the perpetrator’ [64] and avoid victim-blaming language and approaches. Where parents who are victims/survivors are the recipient of interventions, these should strengths-based approach and recognise the parent as I resource for the child, and in particular in providing a secure and supportive relationship to minimise impact upon the child from the IPVA [69]. Finally, all services, and more speifically family courts, should take account of IPVA and take all reasonable steps to avoid retraumatising parents and children who are victims/survivors of IPVA. This should include support from an independent specialist court practitioner who is focused upon the needs of victims [29].

### Strengths and limitations

Our review synthesises a large qualitative literature and our data was not restricted by date of study or publication and it identifies a set of persistent themes that are already evident in past and current literature. However, by including therapeutic and health settings alongside more traditional research settings and the experiences of all family members we have been able to identify and develop themes that are systemic and relevant across a wider range of services and provision. Our work has also drawn on multiple qualitative studies, from a wider range of settings and different countries, including participants with varied ethnicities and ages. In this context the findings of this review provide a broader and yet in depth understanding into the lived experience, perceptions and experiences of different family members together as they engage with IPVA services.

There are a number of specific limitations that need to be recognised. Within the review process, we were reliant and restricted in our own understanding by the qualitative data and quotes that were included in the original publications and studies. We made appropriate efforts to minimise the risk of publication bias, however, a large proportion of studies are not published [90] with striking findings (of very good or very poor service experiences) being more likely to be published [111]. With regards to limitations in the evidence, many of the participants in the study were recalling past and previous experiences. This may increase the risk of recall bias, wherein participants in the original studies could have underestimated or overestimated their (or their children’s) positive or negative experiences and the impact of accessing IPVA services and settings [23]. Despite searching the internation literature, and including studies published in languages other than English, the majority of studies were conducted in the Global North. There is a clear lack of data and perspectives of children (speaking for themselves) and fathers who engaged with IPVA services. Finally, there was very little on the experiences of mothers, children and fathers in relation to impact for those who dropped out of services and had to move on with no support from IPVA services.

## Conclusion

Services should provide a whole-family approach, which responds to the needs both the parent and child victim/survivor, and recognises the parental identity of the perpetrator. Interventions with adult victims/survivors should take a strengths-based approach, whilst holding the perpetrator to account. Particular care is needed when families are involved in family court to avoid re-traumatisation.

## Supplementary Information

Supplementary Material 1.

## Data Availability

Details of includes studies are provided within the manuscript and referenced. Extracted data available on request.
