# Supplementary material for: A systematic review of qualitative studies exploring how parents affected by intimate partner violence and abuse and their children experience child welfare, health and criminal justice responses
Source: BMC Public Health. 2026 Mar 7;26:1822. doi: 10.1186/s12889-026-26838-y (PMC13244937; doi:10.1186/s12889-026-26838-y)
Supplement: Supplementary file 1 — Supplementary Material 1. [file 12889_2026_26838_MOESM1_ESM.docx]

**MEDLINE (OVID)**

1. (Domestic violence or domestic abuse or perpetrator* or emotional abuse or psychological abuse or verbal abuse or economic abuse or Financial abuse).ab,ti.
2. exp Domestic Violence/
3. exp Intimate Partner Violence/
4. (intimate adj3 abuse).ab,ti.
5. (intimate adj3 violence).ab,ti.
6. (Parent* or mother or father or child* or famil*).ab,ti.
7. Parents/px [Psychology]
8. (Social care or social work or welfare services or social services or child welfare or family services or family support or family intervention or troubled families or general practitioner or GP or nurs* or criminal justice or crown prosecution service or court or police or perpetrator programme or men$ programme or probation or offender management or counsel* or intervention or support or therap* or group* or care management or care co*ordination or refuge or maternity or midwi* or primary care or secondary care or emergency care or health*care voluntary or community or third sector or charit*).ab,ti.
9. (perceive or perception or perspective or view or experience or attitude or belief or opinion or feel or know or understand).ab,ti.
10. (Qualitative or qualitative analysis or qualitative research or mixed methods).ab,ti.
11. (Interview or grounded theory or ethnography or interpretative phenomenological analysis or phenomenology or focus group or content analysis or thematic analysis or constant comparative or participant observation).ab,ti.
12. exp Qualitative research/
13. 1 or 2 or 3 or 4 or 5
14. 6 or 7
15. 9 or 10 or 11 or 12
16. 8 and 13 and 14 and 15

**Psyinfo (OVID)**

1. (Domestic violence or domestic abuse or perpetrator* or emotional abuse or psychological abuse or verbal abuse or economic abuse or Financial abuse).ab,ti.
2. exp Domestic Violence/
3. exp Intimate Partner Violence/
4. (intimate adj3 abuse).ab,ti.
5. (intimate adj3 violence).ab,ti.
6. (Parent* or mother or father or child* or famil*).ab,ti.
7. family members/ or family/
8. (Social care or social work or welfare services or social services or child welfare or family services or family support or family intervention or troubled families or general practitioner or GP or nurs* or criminal justice or crown prosecution service or court or police or perpetrator programme or men$ programme or probation or offender management or counsel* or intervention or support or therap* or group* or care management or care co*ordination or refuge or maternity or midwi* or primary care or secondary care or emergency care or health*care voluntary or community or third sector or charit*).ab,ti.
9. exp Health Care Services/
10. exp Social Services/
11. (perceive or perception or perspective or view or experience or attitude or belief or opinion or feel or know or understand).ab,ti.
12. (Qualitative or qualitative analysis or qualitative research or mixed methods).ab,ti.
13. (Interview or grounded theory or ethnography or interpretative phenomenological analysis or phenomenology or focus group or content analysis or thematic analysis or constant comparative or participant observation).ab,ti.
14. exp Qualitative research/
15. 1 or 2 or 3 or 4 or 5
16. 6 or 7
17. 8 or 8 or 10
18. 11 or 12 or 13 or 14
19. 15 and 16 and 17 and 18

**Embase (OVID)**

1. (Domestic violence or domestic abuse or perpetrator* or emotional abuse or psychological abuse or verbal abuse or economic abuse or Financial abuse).ab,ti.
2. (intimate adj3 abuse).ab,ti.
3. (intimate adj3 violence).ab,ti.
4. exp domestic violence/
5. exp partner violence/
6. (Parent* or mother or father or child* or famil*).ab,ti.
7. parent/
8. (Social care or social work or welfare services or social services or child welfare or family services or family support or family intervention or troubled families or general practitioner or GP or nurs* or criminal justice or crown prosecution service or court or police or perpetrator programme or men$ programme or probation or offender management or counsel* or intervention or support or therap* or group* or care management or care co*ordination or refuge or maternity or midwi* or primary care or secondary care or emergency care or health*care voluntary or community or third sector or charit*).ab,ti.
9. health care/
10. social care/
11. criminal justice/
12. (perceive or perception or perspective or view or experience or attitude or belief or opinion or feel or know or understand).ab,ti.
13. (Qualitative or qualitative analysis or qualitative research or mixed methods).ab,ti.
14. (Interview or grounded theory or ethnography or interpretative phenomenological analysis or phenomenology or focus group or content analysis or thematic analysis or constant comparative or participant observation).ab,ti.
15. Qualitative analysis/ or qualitative research/
16. 1 or 2 or 3 or 4 or 5
17. 6 or 7
18. 8 or 9 or 10 or 11
19. 12 or 13 or 14 or 15
20. 16 and 17 and 18 and 19

**ProQuest**

(((abstract(domestic violence OR domestic abuse OR battery OR intimate partner abuse OR intimate partner violence OR perpetrator) OR MAINSUBJECT.EXACT.EXPLODE("Domestic violence")) AND (abstract(mother OR father OR parent) OR abstract(child)) AND (abstract(Social care OR social work OR welfare services OR social services OR child welfare OR family services OR family support OR family intervention OR troubled families OR general practitioner OR GP OR nurs* OR criminal justice OR crown prosecution service OR court OR police OR perpetrator programme OR men* programme OR probation OR offender management OR counsel* OR intervention OR support OR therapy OR group OR care management OR care co ordination OR refuge OR matern* OR midwi* OR primary care OR secondary care OR emergency care OR health care) OR MAINSUBJECT.EXACT.EXPLODE("Social work") OR MAINSUBJECT.EXACT.EXPLODE("Health care") OR MAINSUBJECT.EXACT.EXPLODE("Criminal justice")) AND (abstract(Qualitative OR qualitative analysis OR qualitative research OR mixed methods) OR MAINSUBJECT.EXACT.EXPLODE("Qualitative research"))

**Scopus**

( ( TITLE-ABS-KEY ( Interview OR grounded theory OR ethnography OR interpretative phenomenological analysis OR phenomenology OR focus group OR content analysis OR thematic analysis OR constant comparative OR participant observation ) ) OR ( TITLE-ABS-KEY ( qualitative OR qualitative analysis OR qualitative research OR mixed methods ) ) OR ( TITLE-ABS-KEY ( perceive OR perception OR perspective OR view OR experience OR attitude OR belief OR opinion OR feel OR know OR understand ) ) ) AND ( ( TITLE-ABS-KEY ( Social care OR social work OR welfare services OR social services OR child welfare OR family services OR family support OR family intervention OR troubled families ) ) OR ( TITLE-ABS-KEY ( general practitioner OR GP OR nurs? OR criminal justice OR crown prosecution service OR court OR police OR perpetrator programme OR men? programme OR probation OR offender management OR counsel? ) ) OR ( TITLE-ABS-KEY ( intervention OR support OR therap? OR group? OR care management OR care co?ordination OR refuge OR maternity OR midwi? OR primary care OR secondary care OR emergency care OR health?care voluntary OR community OR third sector OR charit? ) ) ) AND ( ( ( TITLE-ABS-KEY ( Domestic violence OR domestic abuse OR perpetrator* OR emotional abuse OR psychological abuse OR verbal abuse OR economic abuse OR Financial abuse ) OR TITLE-ABS-KEY ( intimate NR3 abuse ) ) ) OR ( TITLE-ABS-KEY ( intimate W/3 abuse ) ) AND ( TITLE-ABS-KEY ( intimate W/3 violence ) ) ) AND ( TITLE-ABS-KEY ( Parent* OR mother OR father OR child* OR famil* ) )

**CINHAL**

S1. XB (Domestic violence or domestic abuse or perpetrator* or emotional abuse or psychological abuse or verbal abuse or economic abuse or Financial abuse) OR XB (intimate N3 violence) OR XB (intimate N3 abuse) OR MJ (domestic violence)

S2. [XB (Parent* or mother or father or child* or famil*)](https://research-ebsco-com.libproxy.ncl.ac.uk/search/results?db=cin20&expanders=concept&limiters=None&searchMode=boolean&sort=relevance&sqId=sq%3A1111a37e-6fad-4ec1-b7ee-9d3334ecfa5a&userDirectAction=true)

S3. [XB (Social care or social work or welfare services or social services or child welfare or family services or family support or family intervention or troubled families or general practitioner or GP or nurs* or criminal justice or crown prosecution service or court or police or perpetrator programme or men* programme or probation or offender management or counsel* or intervention or support or therap* or group* or care management or care co*ordination or refuge or maternity or midwi* or primary care or secondary care or emergency care or health*care voluntary or community or third sector or charit*)](https://research-ebsco-com.libproxy.ncl.ac.uk/search/results?db=cin20&expanders=concept&limiters=None&searchMode=boolean&sort=relevance&sqId=sq%3A3370f916-2880-41b4-951c-0341dfb30a22&userDirectAction=true)

S4. XB (perceive or perception or perspective or view or experience or attitude or belief or opinion or feel or know or understand) OR XB (Qualitative or qualitative analysis or qualitative research or mixed methods) OR XB (Interview or grounded theory or ethnography or interpretative phenomenological analysis or phenomenology or focus group or content analysis or thematic analysis or constant comparative or participant observation) OR MJ (qualitative studies)

S1 AND S2. AND S3. AND S4
